# Supplementary material for: Mad2 Induced Aneuploidy Contributes to Eml4-Alk Driven Lung Cancer by Generating an Immunosuppressive Environment
Source: Cancers (Basel). 2021 Nov 30;13(23):6027. doi: 10.3390/cancers13236027 (PMC8656692; doi:10.3390/cancers13236027)
Supplement: Supplementary file 1 [file cancers-13-06027-s001.zip › cancers-1449921-supplementary.pdf]

Supplementary Figure legends

Supplemental Figure S1. A) Representative FISH images of Alk and Alk+Mad2 tumor cells showing aneuploid cells (yellow circle). DNA: white, chromosome 12 probe (blue), 16 (magenta) and 17 (yellow). B) Representative images of HA-Mad2 expression in tumors and non-tumor areas. Scale bar:100µm. The right image is a magnification of the non-tumor area. C) Representative image of PCNA and Ki67 expression in lung tumors and percentage of $PCNA^{+}$ and $Ki67^{+}$ cells in Alk and Alk+Mad2 groups (n=8). Scale bar: 200µm. D) Representative H&E staining of the lungs in both groups. Scale bar: 1mm. E) Size of the nodules measured in mm^2 in lung tissue of Alk and Alk+Mad2 (n=10) groups. ns, not significant. Two-tailed t-test.

Supplemental Figure S 2. A) Schematic of the bronchoalveolar lavage fluid (BALF) and number of alveolar macrophages in the BALF (Alk; n=10, Alk+Mad2; n=10). B) Representative flow cytometry plots showing $CD45^{+}AquaZombie^{-}aGR1^{-}CD11c^{+}SyglecF^{+}$ alveolar macrophages. C) Level of IL-6 in the BALF measured by ELISA at the indicated dpi. This experiment was performed in duplicate. (Alk n=22; Alk+Mad2, n=21). $*p < 0.05$. Two-tailed t-test.

Supplemental Figure S3. A) Representative flow cytometry plots showing the exclusion of cell debris, doublets and dead cells. Single stains were performed for compensation controls, FMO controls to check for fluorescence spread and isotype controls were used to determine the level of non-specific binding. B) Representative flow cytometry plots of lung tumor and non-tumor areas, stained for CD45, NKp46, CD3, CD49a, CD49b. C-D) Percentage of NKp46+ population in tumors (C) and in non-tumor areas (D) (Alk; n=10, Alk+Mad2; n=6). E-F) Level of IFN-γ in the tumors (E) and non-tumor area (F), expressed by mean pixel density (Alk; n=5, Alk+Mad2; n=5). G-H) Percentage of Ly6G positive cells in tumors (G) and in non-tumor areas (H) of Alk, Alk+Mad2 as well as in normal wild type mice (Alk; n=4, Alk+Mad2; n=4, WT; n=3). $*p < 0.05$, $** p < 0.01$, Two-tailed t-test in B, C, D, E, F and one-way Anova in G. .

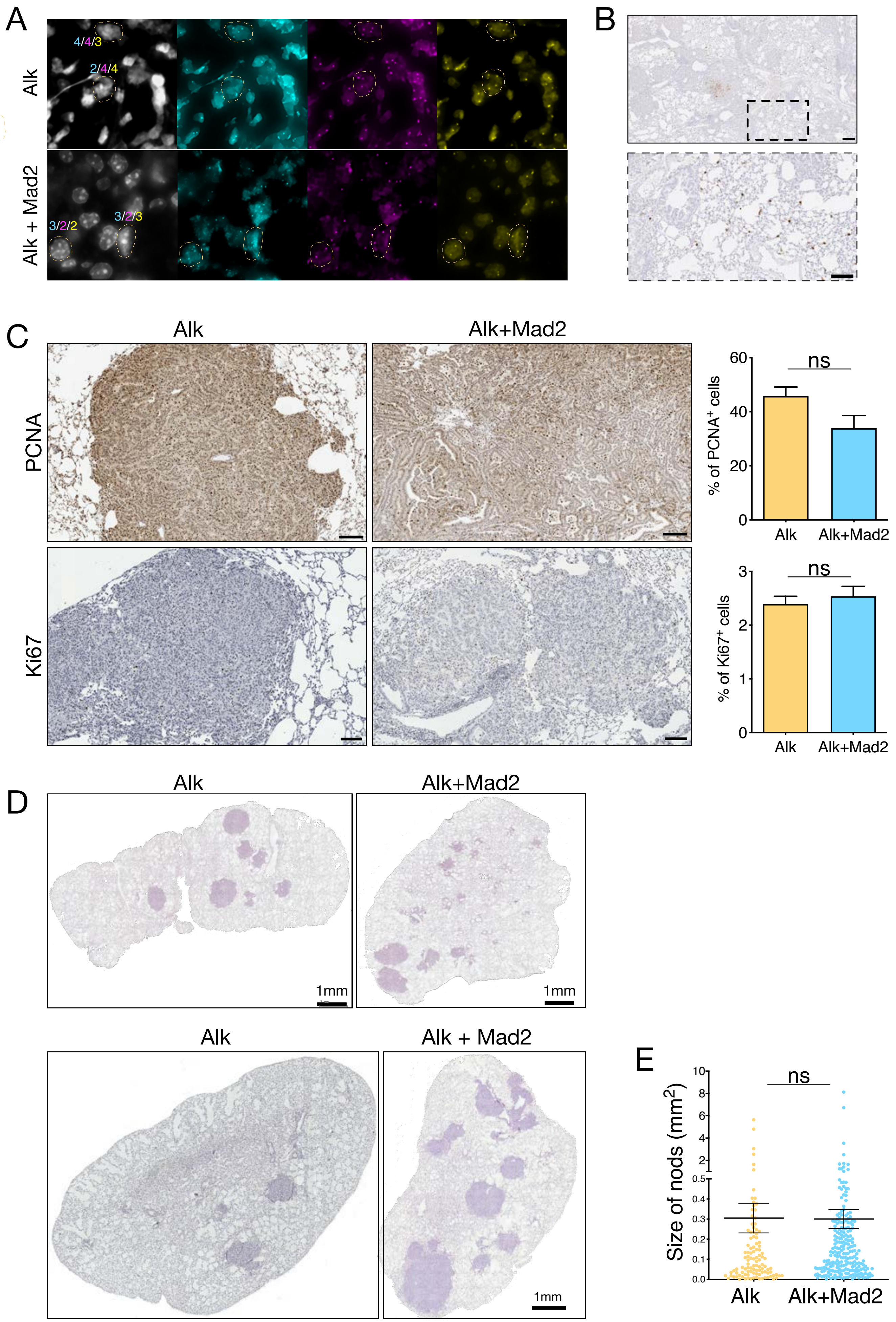

Supplemental Figure S1

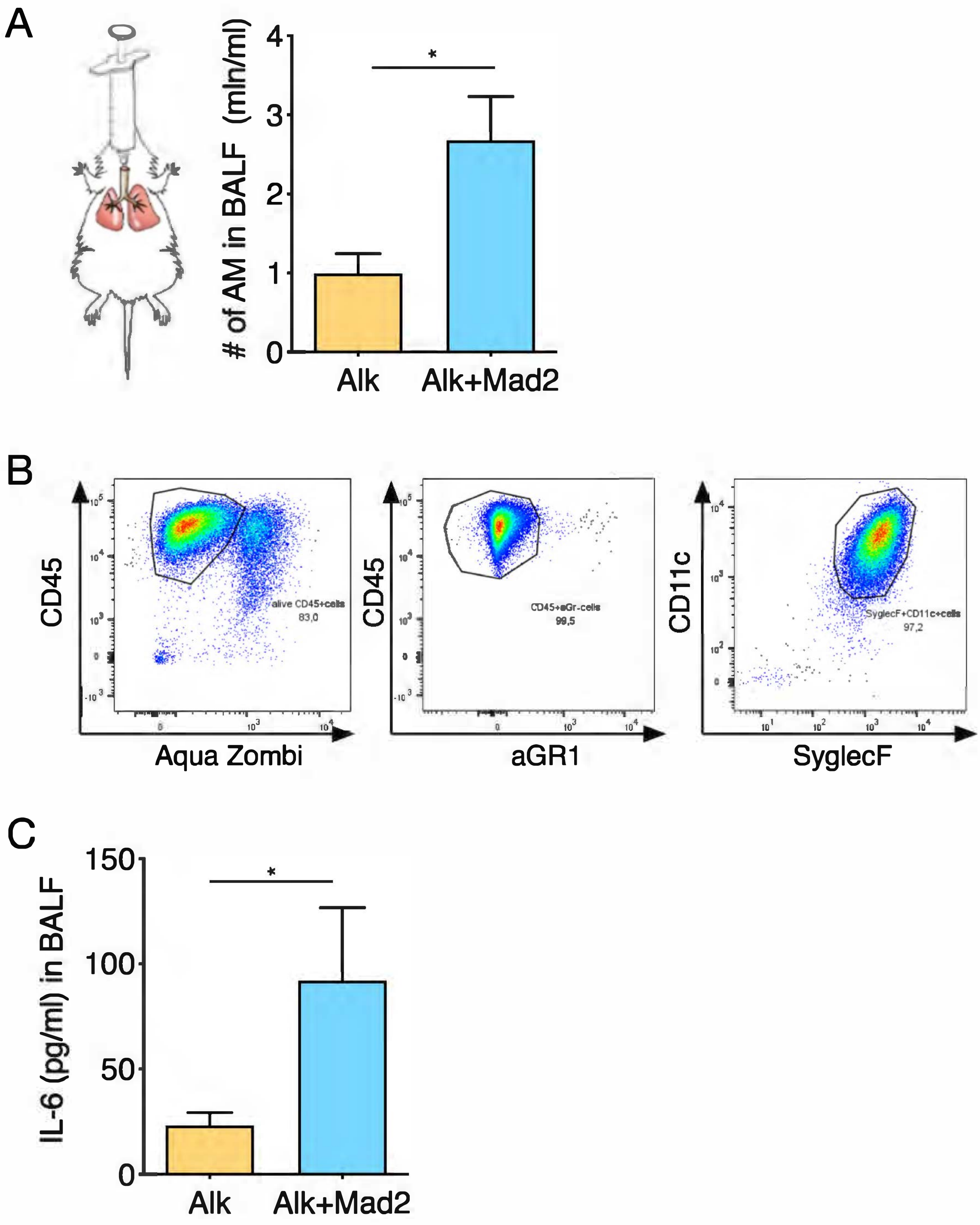

Supplemental Figure S2

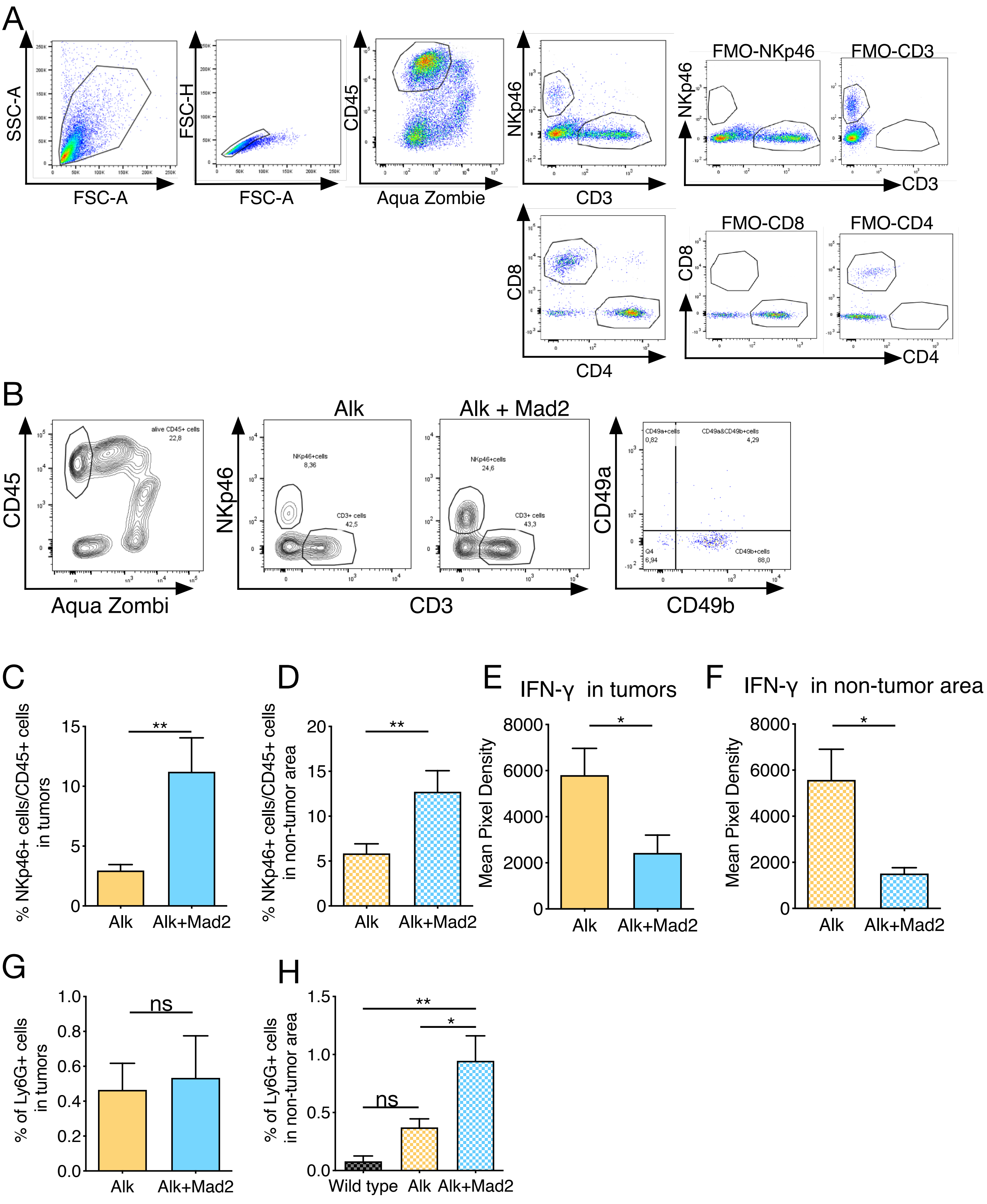

Supplemental Figure S3
